# The Effects of Involuntary Respiratory Contractions on Cerebral Blood Flow during Maximal Apnoea in Trained Divers

**DOI:** 10.1371/journal.pone.0066950

**Published:** 2013-06-26

**Authors:** Troy J. Cross, Justin J. Kavanagh, Toni Breskovic, Petra Zubin Maslov, Mihajlo Lojpur, Bruce D. Johnson, Zeljko Dujic

**Affiliations:** 1 Griffith Health Institute and Heart Foundation Research Centre, Griffith University, Gold Coast Campus, Queensland, Australia; 2 Department of Physiology, University of Split School of Medicine, Split, Croatia; 3 Division of Cardiovascular Diseases, Mayo Clinic, Rochester, Minnesota, United States of America; 4 Department of Anaesthesiology, Clinical Hospital Center Split, Split, Croatia; University of Adelaide, Australia

## Abstract

The effects of involuntary respiratory contractions on the cerebral blood flow response to maximal apnoea is presently unclear. We hypothesised that while respiratory contractions may augment left ventricular stroke volume, cardiac output and ultimately cerebral blood flow during the struggle phase, these contractions would simultaneously cause marked ‘respiratory’ variability in blood flow to the brain. Respiratory, cardiovascular and cerebrovascular parameters were measured in ten trained, male apnoea divers during maximal ‘dry’ breath holding. Intrathoracic pressure was estimated via oesophageal pressure. Left ventricular stroke volume, cardiac output and mean arterial pressure were monitored using finger photoplethysmography, and cerebral blood flow velocity was obtained using transcranial ultrasound. The increasingly negative inspiratory intrathoracic pressure swings of the struggle phase significantly influenced the rise in left ventricular stroke volume (*R* ^2^ = 0.63, *P*<0.05), thereby contributing to the increase in cerebral blood flow velocity throughout this phase of apnoea. However, these contractions also caused marked respiratory variability in left ventricular stroke volume, cardiac output, mean arterial pressure and cerebral blood flow velocity during the struggle phase (*R* ^2^ = 0.99, *P*<0.05). Interestingly, the magnitude of respiratory variability in cerebral blood flow velocity was inversely correlated with struggle phase duration (*R* ^2^ = 0.71, *P*<0.05). This study confirms the hypothesis that, on the one hand, involuntary respiratory contractions facilitate cerebral haemodynamics during the struggle phase while, on the other, these contractions produce marked respiratory variability in blood flow to the brain. In addition, our findings indicate that such variability in cerebral blood flow negatively impacts on struggle phase duration, and thus impairs breath holding performance.

## Introduction

A truly ‘maximal’ apnoea is typified by two distinct, temporal phases. The first phase is a quiescent period where respiratory neuromuscular output is voluntarily suppressed, the glottis is closed, and no movement of the chest wall occurs (i.e., ‘easy-going’ phase) [1]. The beginning of the second period, the ‘struggle’ phase, is marked by the onset of involuntary respiratory contractions that increase in both magnitude and frequency until the breaking point of apnoea is reached [2], [3]. Several factors are known to influence the apnoea breakpoint, these factors include: the degree of hypoxaemia and hypercapnia; lung volume and rate of alveolar gas-exchange; and afferent information originating within the diaphragm during contraction [4]–[7]. In addition to these factors, the ability to maintain adequate delivery of O_2_ to the cerebral tissues must necessarily impact on breath holding performance [8], [9], insofar as brain hypoxia/hypercapnia may induce cognitive impairment, unconsciousness and, in some cases, may result in fatality [10], [11]. The question arises: What mechanisms exist to ensure an adequate supply of O_2_ to the cerebral tissues during maximal apnoea?

It is elementary that maximal breath holding should reduce the O_2_ content of arterial blood [12]. To maintain cerebral O_2_ delivery in spite of this arterial hypoxaemia, bulk blood flow to the brain progressively increases throughout maximal breath holding [8]. The rise in cerebral blood flow during the easy-going phase of apnoea is mediated by: (i) a hypercapnia-induced vasodilation of the cerebral arterioles [13], [14]; and (ii) a progressive increase in mean arterial pressure (MAP) consequent to massive peripheral vasoconstriction [8], [12], [15]. It is of note that the arterial hypertension during the struggle phase appears solely mediated by an increasing left ventricular stroke volume (LVSV) and thus cardiac output (CO) – there is no observable change in total peripheral resistance (TPR) throughout this phase of breath holding [9], [16]. The mechanisms which contribute to the rise in LVSV during the struggle phase remain uncertain.

Previous investigators have hypothesised that forceful inspiratory contractions may act to improve left ventricular output during the struggle phase [9], [16]. Certainly, sustained negative intrathoracic pressure (ITP) is known to improve right venous return and LVSV [17]–[21]. And given that TPR remains unchanged during the struggle phase, any increase in left ventricular output would cause a proportional rise in MAP, therefore contributing to the increase in cerebral blood flow. Although this rationale is attractive, it remains to be shown that involuntary respiratory contractions directly influence left ventricular output during the struggle phase of maximal breath holding.

It must be considered that while involuntary respiratory contractions may facilitate haemodynamics in the manner described above, these contractions are likely to cause pronounced ‘respiratory’ variability in cerebral blood flow during the struggle phase [9]. For example, the acute increases and decreases in ITP which occur throughout the respiratory-cycle are known to impair and augment left ventricular output, respectively [22], [23]. This respiratory-modulation of LVSV causes directionally similar fluctuations in CO and MAP [24], [25], ultimately producing respiratory variability in cerebral blood flow [26], [27]. It can be reasoned that these abrupt variations in cerebral blood flow induce an ongoing mismatch between cerebral tissue O_2_ supply and utilisation which may, in turn, affect cyclic periods of tissue hypoxia in the vicinity of central chemoreceptors. According to this idea, respiratory variability in cerebral blood flow may contribute an additional ventilatory stimulus during apnoea, compounding the individual’s ‘urge to breathe’, directly opposing further apnoeic effort. To date, no study has directly investigated the role of involuntary respiratory contractions in the cerebral blood flow response to maximal breath holding. Moreover, the impact of respiratory variability in cerebral blood flow on apnoea duration (i.e., performance) is unknown.

The first aim of this study was to determine the effects of involuntary respiratory contractions on the cerebral blood flow response to maximal breath holding. It was hypothesised that the increasingly negative ITP swings, generated by inspiratory muscle contraction, would augment LVSV and ultimately contribute to the rise in cerebral blood flow over the struggle phase. At the same time, it was expected that these contractions would produce marked respiratory variability in LVSV, CO, MAP and thus cerebral blood flow during the struggle phase. The second aim of this study was to determine whether cerebral blood flow is a determinant of breath holding performance. It was hypothesised that struggle phase duration would be directly proportional to the rise in bulk cerebral blood flow, yet inversely related to the magnitude of its respiratory variability, throughout this phase of apnoea.

## Methods

### Ethics Statement

The present study conformed to the principles outlined in the Declaration of Helsinki and was approved by the research ethics board at the School of Medicine, University of Split, Croatia.

### Participants and Experimental Design

Ten trained, male apnoea divers (28±2 yr; 183±2 cm, 84±3 kg) volunteered to participate in the study and provided written informed consent. The subjects underwent a pre-participatory health screening to ensure they were physically active non-smokers, with no history of cardiac, pulmonary or metabolic disease. We purposely chose to recruit trained apnoea divers because these individuals are accustomed to extended periods of breath holding. These individuals display, on average, greater absolute durations for the easy-going and struggle phases than naive participants [28].

Subjects first performed a number of pulmonary function tests in the supine position, after which they were instructed to remain supine for 10 mins before performing breath-hold manoeuvres (i.e., rest period). Breath-hold manoeuvres commenced after full inflation to total lung capacity (TLC) while wearing a nose-clip. The subjects were instructed to keep their glottis closed during each breath-hold. Subjects were asked to refrain from performing preparatory hyperventilation, and were not allowed to perform glossopharyngeal insufflations prior to the manoeuvre. Subjects completed two practice trials, followed by 2–3 experimental breath holds, separated by at least 10 mins of supine rest. The subjects were encouraged to completely relax their respiratory musculature during the early period of the breath-hold (i.e., easy-going phase) and, should the urge arise, to allow respiratory contractions to develop “*naturally”*. The subjects were also instructed to avoid contraction of their peripheral musculature, including neck and facial muscles. The latter point was particularly important for obtaining high-quality measurements of middle cerebral artery blood flow velocity using the transcranial Doppler probe (see below). In the event that peripheral muscle activation was observed by the investigator, or reported by the subject, breath holding was immediately terminated. Subjects were excluded from the study if, after a further two attempts, a satisfactory apnoea could not be obtained.

### Pulmonary Function, Arterial O_2_ Saturation and Respiratory Pressures

Before each experiment, the subjects performed a forced vital capacity (FVC) manoeuvre while in the supine posture (Quark PFT, Cosmed, Rome, Italy). All pulmonary function testing was performed and reported in accordance with the American Thoracic Society guidelines [29]. Arterial O_2_ saturation of haemoglobin (SaO_2_) was measured via finger pulse oximetry (Poet II, Criticare Systems, Waukesha, WI). Oesophageal pressure was measured using a latex balloon-tip catheter (Ackrad Laboratories, CooperSurgical, Trumbull, CT, USA) advanced through the nose into the oesophagus [30]. The catheter balloon was then inflated with 1 ml of air, and the “occlusion” test was performed to ensure correct placement of the catheter in lower third of the oesophagus [31]. The catheter was connected to a differential pressure transducer (PX138-005D5V, Omega Engineering Inc., Stamford, CT, USA). The transducer was calibrated before each test using a digital manometer (HHP-90, Omega Engineering inc., Stamford, CT, USA).

### Arterial Blood Pressure and Cerebral Blood Flow Velocity

Beat-by-beat arterial blood pressure was measured using a pneumatic cuff placed around the middle phalanx of the non-dominant hand, and was connected to a photoplethysmograph (Finometer, Finapress Medical Systems, Arnhem, Netherlands). The hand bearing the finger cuff was positioned at the level of the heart in order to negate hydrostatic pressure artifact. The hand was kept in this position for the duration of the experimental protocol. Cerebral blood flow velocity (CBFV) was obtained via transcranial Doppler ultrasound of the proximal middle cerebral artery, measured using a 2-MHz probe fixed at a constant angle over the right posterior temporal “window” (Transcranial Doppler, Multigon, Yonkers, NY, USA).

### Data Analysis and Processing

Oesophageal pressure, finger arterial pressure, and transcranial Doppler flow velocity signals were sampled continuously at 1000 Hz (Powerlab 16SP, ADInstruments Inc., Castle Hill, Australia) and stored on a personal computer for off-line analyses. The oesophageal pressure signal was taken to represent intrathoracic pressure (ITP). The beginning of the struggle phase was identified as the first swing in ITP greater than ±2 cmH_2_O of the mean ITP calculated from the previous 5 s of data. The magnitudes of static inspiratory and expiratory contractions were determined as the minima and maxima of the ITP tracing, respectively. The total time elapsed between two consecutive inspiratory efforts (Ttot) was used to calculate the frequency of respiratory contraction (*f*R = 60/Ttot).

Mean arterial pressure (MAP) and CBFV were computed as the arithmetic means of the arterial pulse wave and transcranial Doppler flow velocity signals over each beat interval, respectively [32]. Left ventricular stroke volume (LVSV) was computed from the arterial pulse wave using the improved Modelflow algorithm [33]. Cardiac output (CO) was taken as the product of heart rate (HR) and LVSV, and total peripheral resistance (TPR) as MAP/CO. The cerebrovascular resistance index (CVRi) was taken as MAP/CBFV. The recorded ITP signal was decimated to 50 Hz. The beat-by-beat time series of HR, LVSV, CO, MAP, TPR, CBFV and CVRi were resampled to 50 Hz using cubic spline interpolation. These haemodynamic variables are under constant, dynamic autonomic control; processes that are most effective within the low-frequency range (i.e., 0.04–0.15 Hz) [27], [34]. Furthermore, these variables are also affected either directly or indirectly by respiration – such modulation of haemodynamics occurs within the broadband of respiratory frequencies (i.e., >0.15 Hz). To extract the variability in each dataset that is due solely to respiratory effort, all time series were band-pass filtered using an 8^th^ order, zero-phase Butterworth filter with the low and high cut-off frequencies set equal to 0.20 and 0.80 Hz. This specific range of frequencies encompassed the upper- and lower-most values for *f*R observed during the experiment in all subjects.

**Figure 1:**
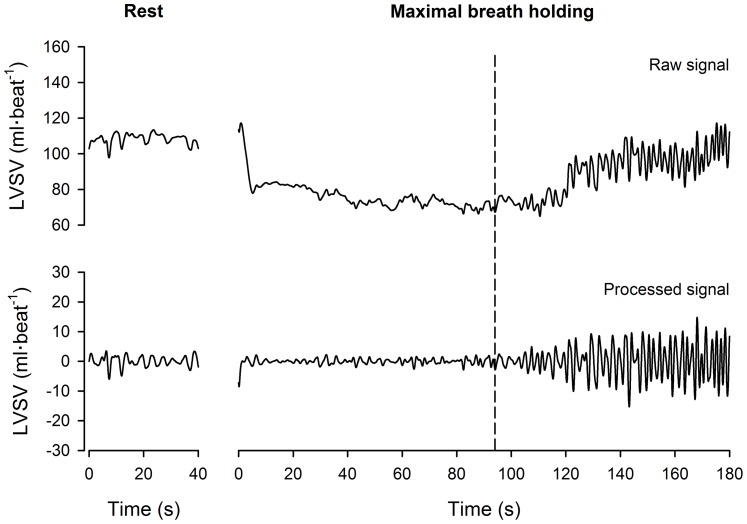
Example of left ventricular stroke volume time-series of a representative subject. The left and right panels display data obtained while the subject was resting supine, and during maximal apnoea, respectively. LVSV: left ventricular stroke volume. The vertical dashed line represents the start of the struggle phase. The raw LVSV tracing was obtained by re-sampling beat-by-beat values to 50 Hz via cubic spline interpolation. The interpolated data was band-pass filtered using an 8^th^ order, zero-phase Butterworth filter with low and high cut-off frequencies of 0.20 and 0.80 Hz (i.e., the broadband of respiratory frequencies). This ‘processed’ data was taken to represent the respiratory variability in LVSV.

The above parameters were averaged into seven time-epochs representing: the resting period; and the first, second and third tertiles of the easy-going phase (EP1, EP2 and EP3) and the struggle phase durations (SP1, SP2 and SP3), respectively. The means (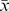) of each time series were computed using raw data, while standard deviations (*SD*) were calculated using the filtered (‘processed’) time series (**Figure 1**). The respiratory variability in each time-series was quantified by the coefficient of variation: *CV* (%) = 100(*SD*/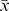). The absolute *SD* of the ITP time-series was used as an index of respiratory variability, because *CV* cannot be computed for data where 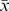 may approach zero. Lastly, respiratory variability was not calculated for TPR and CVRi. We acknowledge that previous investigators have reported high-frequency variability in TPR and CVRi in humans [32]. However, within the scope of the present research design, we could not properly discern whether such variability in TPR and CVRi was of physiological origin or simply reflected computational errors induced by slight temporal delays between measurement devices (e.g., MAP measured via the photoplethysmograph and CBFV obtained via the Doppler ultrasound probe).

### Statistical Analyses

All data were averaged across repeated trials. One-way analyses of variance were used to evaluate the main-effect of time (rest, EP1, EP2, EP3, SP1, SP2 and SP3) on respiratory, cardiovascular and cerebrovascular variables. Pair-wise comparisons were assessed using the Bonferroni *post-hoc* adjustment. The role of inspiratory and expiratory ITP swings in the augmentation of LVSV during the struggle phase was examined using stepwise multiple linear regression. Path analysis was used to determine the extent to which respiratory contractions affect respiratory variations in CBFV during the struggle phase (see below). Lastly, the influence of cerebral blood flow on breath holding performance was assessed using stepwise multiple linear regression, where the overall rise and magnitude of respiratory variability in CBFV during the struggle phase were input into the regression model as independent variables, while the duration of the struggle phase (in seconds) was input as the dependent variable. Results are presented as mean ± standard error of the mean (*SEM*) unless stated otherwise. All data were analysed using SPSS 20.0 (SPSS, Inc., Chicago, IL, USA). Statistical analyses were considered significant if *P*<0.05.

### Path Analysis

**Figure 2:**
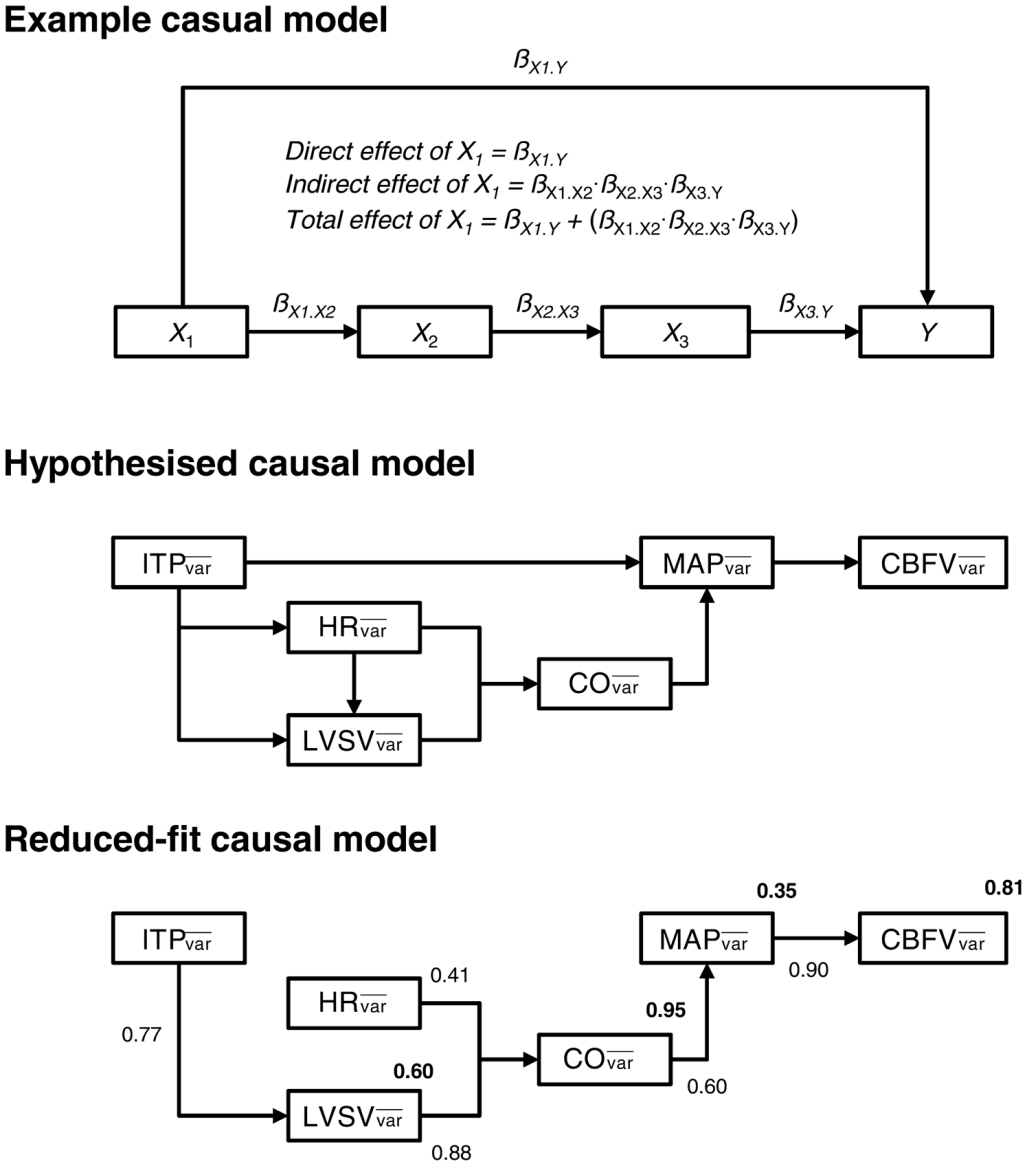
Path analysis. 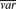
: mean variability observed in the corresponding parameter during the struggle phase; ITP: intrathoracic pressure; HR: heart rate; LVSV: left ventricular stroke volume; CO: cardiac output; MAP: mean arterial blood pressure; TPR: total peripheral resistance; CVRi: cerebrovascular resistance index; CBFV: cerebral blood flow-velocity. The top panel depicts a hypothetical Path model to illustrate the direct and indirect effects of each parameter (*X*_1_, *X*_2_ and *X*_3_) on the terminal endogenous variable (i.e., *Y*). The symbol *ß* represents the standardised path coefficient describing the influence of one variable on another. See Methods for further explanation. The hypothesised causal model used to examine the influence of respiratory contractions on the respiratory variability in cerebral blood flow velocity is displayed in the middle-panel. The ‘reduced-fit’ causal model is presented in the bottom-panel. Only those parameters which were identified as significant (*P*<0.05) mediators of the terminal dependent variable (i.e., 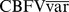) are retained in this panel. Solid arrows denote the direction of causality between corresponding parameters. Values located adjacent to arrows represent the standardised coefficient of the corresponding path. Values displayed in bold represent the coefficient of determination (*R* ^2^) of the relationship between the corresponding parameter and all preceding paths.

Path analysis is used to determine whether a set of multivariate data are consistent with a conceptual structural model [35], [36]. The structural model theorises a number of causal relationships between variables, which may be either *direct* or *indirect* in nature. The influence of one variable on another is determined by performing a series of multiple and simple linear regression analyses which, in turn, yield a set of standardised *ß*-coefficients for each directed path [35]–[37]. A *ß*-coefficient of –0.50 indicates that for every 1 *SD* increase in variable *X*_1_ there occurs a 0.50 *SD* decrease in variable *Y*. Conversely, a *ß*-coefficient of 1.25 indicates that for every 1 *SD* increase in variable *X*_1_ there occurs a 1.25 *SD* increase in variable *Y.* In the example model displayed in **Figure 2**, *ß*_X1.Y_ represents the *direct* effect of *X*_1_ on *Y*. It is also possible that the effect of *X*_1_ on *Y* is mediated by other intervening variables separate from the direct path *X*_1_ *→Y*. For example, it may be that: *X*_1_ *→X*_2_ *→X*_3_ *→Y*. The total *indirect* effect of *X*_1_ on Y is then quantified by summing the products of *ß*-coefficients for all mediating or intervening paths (e.g., *ß*_X1.X2_ ⋅ *ß*_X2.X3_ ⋅ *ß*_X3.Y_). The total impact of *X*_1_ on the terminal dependent variable Y is given by the sum of its direct and indirect effects. Path analysis was conducted using SPSS AMOS 20.0 (SPSS, Inc., Chicago, IL, USA). The causal model used in this analysis was constructed *a priori*, using the rationale outlined below.

The multivariate data used in the hypothesised causal model depicted in **Figure 2** were the mean variability (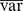) in respiratory, cardiovascular and cerebrovascular parameters during the struggle phase. The majority of the paths outlined in this model were justified for inclusion because of the well-known physiological dependencies between parameters. The remaining paths on the diagram were justified based on the following rationale: Path 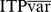 to 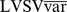: during tidal inspiration, the fall in ITP abruptly impairs left ventricular output and decreases LVSV, while the expiratory rise in ITP augments LVSV [22], [23]. These “within-breath” changes represent the respiratory modulation of LVSV in healthy individuals; Path 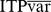 to 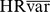: it is known that some of the variability in spontaneous HR is attributable to central respiratory rhythm (respiratory sinus arrhythmia) [24], [25] and may be evidenced by synchronous variations in ITP and HR; Path 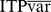 to 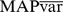: changes in ITP may be transmitted through the wall of the thoracic aorta, directly modulating arterial blood pressure [38].

## Results

### Pulmonary Function, Arterial O_2_ Saturation and Breath Holding Performance

The resting pulmonary function of our participants appeared within the normal range, where FVC, forced expiratory volume in 1 s (FEV_1_) and FEV_1_/FVC were 6.57±0.36 L (120±5% predicted), 5.40±0.25 L (118±4% predicted) and 0.82±0.01 (100±1% predicted), respectively. The subjects’ average breath-hold duration was 187±7 s. The coefficient of variation (*CV*) for total breath-hold duration was 10±2%, indicating minor variability between repeated breath holdings efforts. The mean durations of the easy-going and struggle phases were 96±4 s (51±2% of total) and 91±5 s (49±2% of total), respectively. All subjects were able to produce at least two satisfactory apnoeic efforts without discernable activation of their peripheral musculature. Moreover, our subjects did not experience loss of consciousness, or overt neurological impairment during and after maximal apnoea.

All subjects displayed significant (*P*<0.05) arterial desaturation during the breath-hold manoeuvre, where SaO_2_ values decreased from rest (99±1%) to a nadir (87±2%) which occurred ∼30 s after the end of breath holding – this delay represents the circulatory transit time from the lungs to the finger where the oximeter probe was located.

### Respiratory, Cardiovascular and Cerebrovascular Parameters During Breath Holding

**Figure 3:**
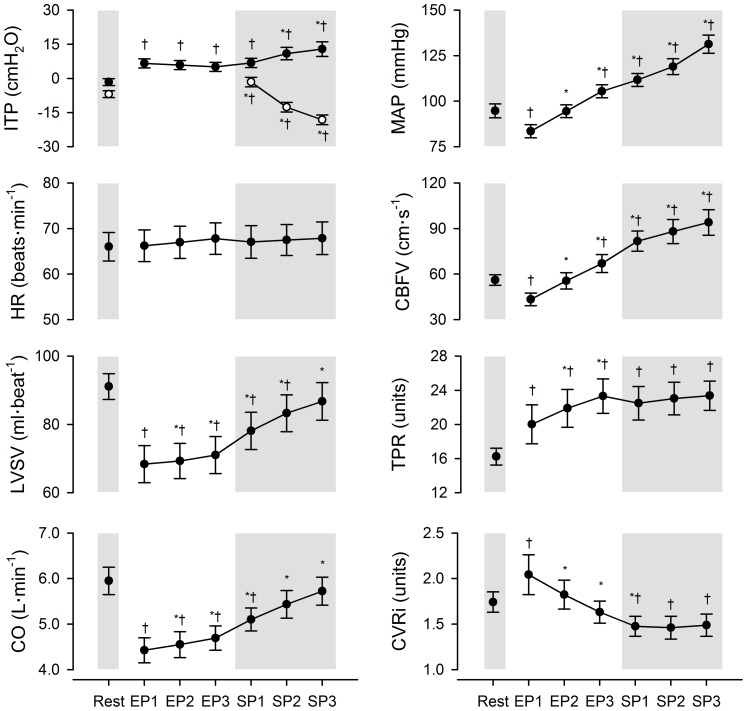
Time-course changes in respiratory, cardiovascular and cerebrovascular variables at rest and during maximal apnoea. Vertical error bars represent means ± *SEM*. EP1, EP2 and EP3: first, second and third tertiles of the easy-going phase; SP1, SP2 and SP3: first second and third tertiles of the struggle phase; ITP: intrathoracic pressure; HR: heart rate; LVSV: left ventricular stroke volume; CO: cardiac output; MAP: mean arterial blood pressure; CBFV: cerebral blood flow-velocity; TPR: total peripheral resistance in units of mmHg⋅L^−1^⋅min; CVRi: cerebrovascular resistance index in units of mmHg⋅cm^−1^⋅s. The closed and open circles in the top left-hand panel represent expiratory and inspiratory ITP swings, respectively. ^*^Significantly different from previous time-point, *P*<0.05. ^†^Significantly different from resting value, *P*<0.05.

The time-course changes in respiratory and haemodynamic variables during maximal breath holding are displayed in **Figure 3**. HR did not change significantly from resting values through breath holding. LVSV and CO decreased below resting values at the beginning of the easy-going phase (*P*<0.05). After this initial decline, LVSV and CO progressively increased with time (*P*<0.05) reaching values similar to those observed at rest by the breakpoint. MAP and CBFV dropped below resting values at the start of the easy-going phase (*P*<0.05), rising with time thereafter until the apnoea breakpoint (*P*<0.05). TPR increased abruptly at the onset of breath holding, achieving its highest value by the end of the easy-going phase (*P*<0.05), whereafter TPR remained stable above baseline until the end of breath holding (*P*<0.05). CVRi was notably higher than resting values at the start of the easy-going phase (*P*<0.05), but progressively decreased from this point until the middle of the struggle phase (*P*<0.05).

At the onset of involuntary respiratory contractions, inspiratory ITP swings became more negative with time from the beginning until the end of the struggle phase (*P*<0.05). Expiratory ITP swings significantly increased from the middle of the struggle phase until the apnoea breakpoint (*P*<0.05). The frequency of contractions (*fR*) increased from the beginning until the end of the struggle phase (SP1 = 20±5 efforts•min^−1^, SP2 = 25±4 efforts•min^−1^, SP3 = 34±4 efforts•min^−1^; All *P*-values were <0.05). The values for *f*R were significantly higher during this phase of breath holding than those observed at rest (12±4 efforts•min^−1^, *P*<0.05).

### The Influence of Intrathoracic Pressure on Left Ventricular Stroke Volume During the Struggle Phase

**Figure 4:**
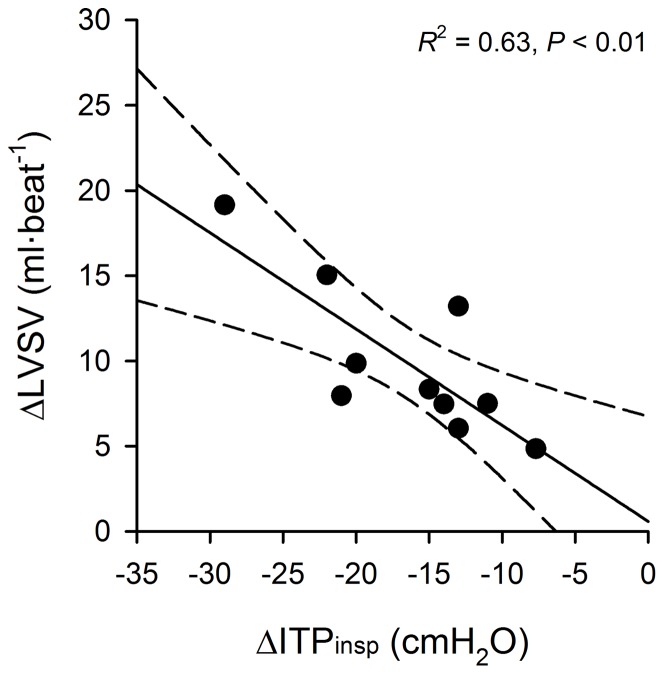
Influence of inspiratory intrathoracic pressure on left ventricular stroke volume during the struggle phase. LVSV: left ventricular stroke volume; Δ: absolute change in values from beginning until the end of the struggle phase of breath holding; ITPinsp: inspiratory swing in intrathoracic pressure during involuntary respiratory contractions. The solid line represents the line of best-fit determined from simple linear regression. The hashed lines denote the ±95% confidence interval of the regression function.

Stepwise multiple regression revealed that the increasing magnitude of inspiratory (not expiratory) ITP swings influenced the rise in LVSV during the struggle phase of maximal breath holding (**Figure 4**, *R* ^2^ = 0.63, *P*<0.01). The linear equation representing this relationship was: ΔLVSV = –0.565•ΔITPinsp +0.580, where ΔITPinsp is the change in the magnitude of inspiratory ITP swings from the beginning until the end of the struggle phase.

### The‘Respiratory’ Variability in Respiratory and Haemodynamic Parameters During Breath Holding

**Figure 5:**
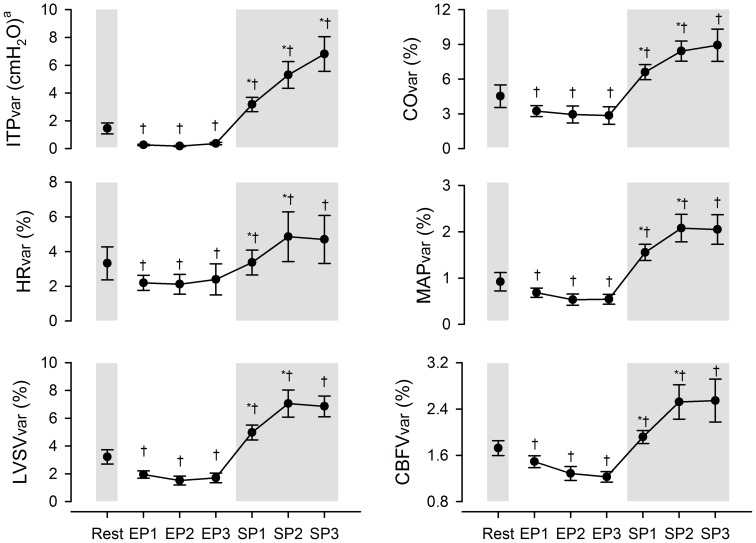
Respiratory variability in intrathoracic pressure, cardiovascular and cerebrovascular parameters at rest and during maximal apnoea. Vertical error bars represent mean coefficients of variation (*CV*) ± *SEM*. EP1, EP2 and EP3: first, second and third tertiles of the easy-going phase; SP1, SP2 and SP3: first second and third tertiles of the struggle phase; ITP: intrathoracic pressure; HR: heart rate; LVSV: left ventricular stroke volume; CO: cardiac output; MAP: mean arterial blood pressure; CBFV: cerebral blood flow velocity; TPR: total peripheral resistance; CVRi: cerebrovascular resistance index. ^a^The standard deviation of ITP is presented here as a measure of respiratory variability instead of *CV* (see Methods). ^*^Significantly different from previous time-point, *P*<0.05. ^†^Significantly different from resting value, *P*<0.05.

The respiratory variability in all parameters was systematically lower during the easy-going phase compared with resting values (**Figure 5**; *P*<0.05). Thereafter, the variability in each parameter increased from the start until the middle of the struggle phase (*P*<0.05) – 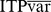 was the only variable that continued to rise until the apnoea breakpoint (*P*<0.05). The overall coefficient of determination (*R* ^2^) for the entire ‘reduced-fit’ causal model displayed in **Figure 2** was 99% (*P*<0.05). The direct, indirect and total effects of each parameter on 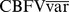 are reported in **Table 1**. It was revealed that 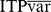 exerted a modest and positive influence on the respiratory variability in cerebral blood flow during the struggle phase (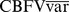). The indirect path through which 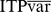 affected 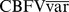 was mediated by 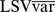, 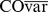, and finally, 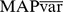. 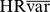 exerted the least overall influence on 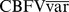.

**Table 1:** The impact of‘respiratory’ variability in intrathoracic pressure and central haemodynamics on cerebral blood flow velocity during the struggle phase.

	Input	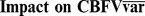		
	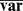 (%)	Direct	Indirect	Total
ITP	5.5±0.8	–	0.366	0.366
HR	3.3±0.4	–	0.219	0.219
LVSV	5.9±0.5	–	0.474	0.474
CO	7.6±0.5	–	0.536	0.536
MAP	1.8±0.2	0.901	–	0.901

Values represent standardised *ß*-coefficients obtained from the ‘reduced-fit’ model depicted in **Figure 2**. 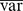: mean variability (coefficient of variation in percent) observed in the corresponding parameter during the struggle phase of breath holding; ITP: intrathoracic pressure; HR: heart rate; LVSV: left ventricular stroke volume; CO: cardiac output; MAP: mean arterial blood pressure; CBFV: cerebral blood flow velocity. ^a^The standard deviation of ITP is presented here as a measure of respiratory variability in place of the coefficient of variation (see Methods). The group-average for 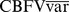 used in the above Path analysis was 2.2±0.1%.

### The Influence of Cerebral Blood Flow on Struggle Phase Duration

**Figure 6:**
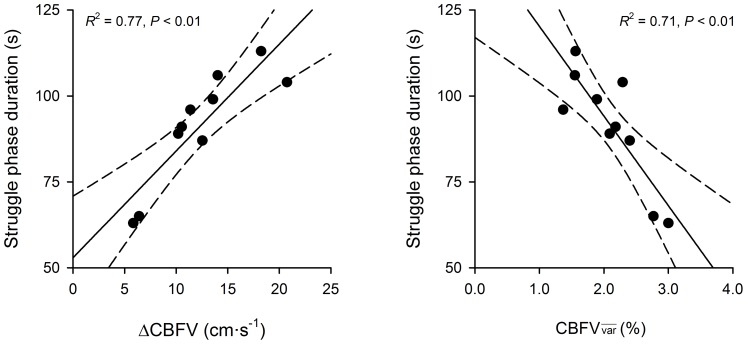
The influence of cerebral blood flow velocity on the struggle phase duration. Δ: absolute change in values from beginning until the end of the struggle phase of breath holding; CBFV: cerebral blood flow velocity; 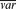: mean variability observed in the corresponding parameter during the struggle phase. The *R* ^2^ values presented in these panels are calculated from each independent variable’s zero-order correlation coefficient. The solid lines represents the line of best-fit determined from simple linear regressions. The hashed lines denote the ±95% confidence intervals of the regression functions.

Stepwise multiple regression revealed that the rise in bulk cerebral blood flow (ΔCBFV) and 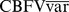 during the struggle phase significantly influenced the duration of this phase of apnoea itself (**Figure 6**; *R* ^2^ = 0.97, *P*<0.01). The equation describing this multiple linear relationship was: SPD = (2.12•ΔCBFV) – (16.19•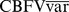) +99.27, where SPD is the duration of the struggle phase in seconds. Semi-partial correlation coefficients were obtained from the stepwise multiple regression. These coefficients represent the strength of influence of one independent variable on the dependent variable, after the variance due to other independent variables has been partialled out [39]. The semi-partial correlation coefficients for the input parameters ΔCBFV and 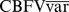 were 0.507 and –0.433, respectively.

## Discussion

The present study demonstrates that involuntary respiratory contractions significantly influence the rise in CBFV via facilitation of LVSV during the struggle phase of breath holding. At the same time, these contractions generate abrupt oscillations in central haemodynamics, ultimately producing marked ‘respiratory’ variability in CBFV during the struggle phase (i.e., 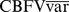). Moreover, this study is the first to provide evidence that a greater magnitude of 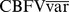 impairs breath holding performance in man. These findings provide further insight into the effects of involuntary respiratory contractions on the cerebral blood flow response to maximal apnoea, and on breath holding performance.

### Cerebral Blood Flow at the Onset of Maximal Breath Holding

LVSV, CO and MAP together decreased at the onset of maximal breath holding (**Figure 3**), consistent with previous observations [8], [9], [16]. Two possible reasons exist which may explain this abrupt impairment of central haemodynamics at the onset of maximal apnoea. Firstly, the large, negative inspiratory ITP swing generated immediately prior to breath holding at TLC may transiently enhance right ventricular preload and, subsequently, reduce LVSV via ventricular interdependence [40]. Secondly, after breath holding commences, the positive elastic “recoil” pressures exerted by the lungs and chest wall at TLC [41] may increase left ventricular afterload, thereby continuing to impair central haemodynamics throughout the early moments of apnoea (i.e., EP1) [42]. Importantly, we observed that CVRi increased from rest to the beginning of maximal apnoea, possibly in an effort to maintain CBFV despite the marked fall in MAP (i.e., cerebral autoregulation) [27]. Nevertheless, the magnitude of rise in CVRi at this time did not compensate for the observed reduction in arterial ‘driving’ pressure and, consequently, CBFV immediately decreased below resting values at the start of apnoea.

### Cerebral Blood Flow During the Easy-going Phase

The asphyxia induced by prolonged apnoea results in vasodilation of the cerebral vasculature and a mounting arterial hypertension during the easy-going phase of breath holding [8], [13]–[15]. These haemodynamic responses together favour a continuous rise in cerebral blood flow [8], [14]. The arterial hypertension during the easy-going phase is primarily due to chemoreflex activation of the sympathetic nervous system, resulting in a massive vasoconstriction of the peripheral vasculature [15], [43]. Our data are in agreement with the above, wherein the subjects of the present study displayed a progressive decline in CVRi and a continuous rise in TPR, MAP and CBFV over the easy-going phase. It is of interest to note that LVSV systematically increased from the beginning until the end of the easy-going phase in our subjects. This progressive rise in LVSV affected an overall increase in systemic CO, thereby contributing to the observed arterial hypertension. It has been hypothesised that the massive peripheral vasoconstriction during maximal apnoea promotes a central-shift in blood volume, which may augment right venous return and, subsequently, left ventricular output [8], [44]. Notwithstanding this potential increase in central blood volume, the contribution of CO to the mounting arterial hypertension during the easy-going phase was relatively minor compared to the direct effect on MAP exerted by peripheral vasoconstriction.

### Cerebral Blood Flow During the Struggle Phase

In the present study, CBFV continued to increase over the duration of the struggle phase, as noted previously by others [8], [16]. It has long been known that arterial CO_2_ is a potent regulator of bulk cerebral blood flow, wherein arterial hypercapnia and hypocapnia elicit cerebral vasodilation and vasoconstriction, respectively [45]. It should be noted that cerebral arterioles are more responsive to changes in arterial CO_2_ than to sympathetic activation [46]. Thus, while measurements of arterial CO_2_ were not obtained in the present study, its effects on bulk cerebral blood flow may be inferred via changes in CVRi. On this point, the fall in CVRi during the struggle phase was relatively minimal compared with the rise in MAP (i.e., –1% v 18%). Thus, we propose that MAP was the primary mediator of the rise in bulk cerebral blood flow during this phase of breath holding.

The mounting arterial hypertension during the struggle phase could not be explained by a further increase in TPR – a finding consistent with that previously reported from our laboratory [9], [16]. It is possible that the magnitude of peripheral tissue hypoxia and/or hypercapnia at this time directly opposed further vasoconstriction, despite increasing sympathetic vasoconstrictor drive [15], [43]. Accordingly, the rising MAP and, by extension, CBFV were primarily due to an increasing systemic CO. Previous investigators have hypothesised that involuntary respiratory contractions may, at least in part, explain the progressive augmentation of left ventricular output during the struggle phase [9], [16].

Due to the location of the heart within the thorax, changes in ITP are directly transmitted to the right atria [18], [19]. In the present study, the increasingly negative inspiratory ITP swings during the struggle phase most likely decreased right atrial pressure, favouring venous return to the right heart [17]. Certainly, recent evidence suggests that venous blood flow through the inferior vena cava abruptly increases at the onset of respiratory contractions during breath holding [16]. Our data are consistent with the above hypothesis in that 63% of the variance observed in ΔLVSV could be accounted for by the increasing negativity of inspiratory efforts during the struggle phase (i.e., ΔITPinsp). Stated in other words, the more negative that inspiratory ITP swings became during the struggle phase, the larger was the increase in LVSV (see **Figure 3**). To the best of our knowledge, this is the first study to clearly demonstrate that the augmenting CO during the struggle phase is related to a ‘respiratory-mediated’ facilitation of left ventricular output.

### ‘Respiratory’ Variability in Cerebral Blood Flow During the Struggle Phase

During tidal inspiration, the increasing negativity of ITP transiently impairs left ventricular filling (decreased preload) and emptying (increased afterload), thereby causing an abrupt fall in LVSV – the converse is true during tidal expiration [22], [23]. Such respiratory-synchronous modulation of left ventricular output produces respiratory variability in MAP and cerebral blood flow [24]–[27]. Given that respiratory effort directly modulates ITP, it is perhaps not surprising that 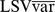, 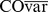, 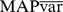 abruptly increased at the beginning of the struggle phase. Moreover, Path analysis revealed that 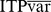 indirectly mediated 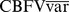 via 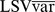 throughout the struggle phase. These results provide strong evidence that our values for 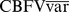 represented the haemodynamic effects of involuntary respiratory contractions, and were not influenced by other physiological processes operating in the same passband of frequencies (i.e., 0.20 to 0.80 Hz).

### Cerebral Blood Flow and Breath Holding Performance

It can be reasoned that respiratory variability in cerebral blood flow and, by extension, O_2_ delivery during this phase induces transient mismatching between cerebral tissue O_2_ supply and utilisation. Cerebral tissue oxygenation would therefore vary in phase with ITP, evidencing regional tissue hypoxia and hyperoxia during static inspiratory and expiratory efforts, respectively. Recent observations made by Dujic *et al*. [9] support this thesis. These investigators reported that phasic oscillations in cerebral tissue oxygenation spontaneously emerge at the onset of involuntary respiratory contractions during breath holding in elite apnoea divers.

One may speculate that repetitive periods of cerebral hypoperfusion may affect local hypoxia in the vicinity of central chemoreceptors, constituting a ventilatory stimulus that is eventually perceived as breathlessness [47], [48]. Accordingly, those individuals who display greater respiratory variability in cerebral blood flow may also experience a heightened sensation of breathlessness during the struggle phase. The augmented dyspnoea would directly oppose apnoeic effort in these individuals, impairing breath holding performance. An alternative explanation for the inverse relation between 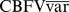 and struggle phase duration is that increasing respiratory pressure-development, as occurs during this phase of breath holding [2], [3], necessitates an increase in energetic expenditure of the respiratory muscles. In turn, the progressively augmenting respiratory muscle O_2_ consumption may therefore compound the fall in O_2_ delivery to the cerebral tissues during the struggle phase, impairing overall breath holding performance in a manner that is proportional to 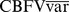. Nevertheless, we demonstrate here, for the first time, that struggle phase duration is directly proportional to ΔCBFV yet inversely related to 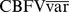. Moreover, the semi-partial *R* ^2^ values for ΔCBFV and 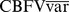 were 26% and 19%, respectively, indicating that the independent effects of each parameter on struggle phase duration were similar in strength, though opposite in direction. In turn, it may be stated that while a greater rise in bulk cerebral blood flow during the struggle phase facilitates longer breath holding, a larger amount of respiratory variability in cerebral blood flow negatively impacts on breath holding performance in man. Further studies are required to validate the cause-and-effect nature of this relationship.

### Methodological Considerations

Transcranial Doppler ultrasound of the right middle cerebral artery (MCA) was used in the present study to estimate bulk cerebral blood flow during maximal breath holding [49]. This technique does not measure volumetric blood flow *per se*, rather it quantifies the velocity of blood passing through the insonated artery [50]. Changes in the diameter of the insonated artery may therefore alter blood velocity, independent of volumetric flow through the MCA. However, the weight of available evidence suggests that MCA diameter is relatively stable over a wide range of MAP and arterial blood gases [51], [52]. To maximise the length of apnoea in our subjects, each breath-hold manoeuvre was initiated from total lung capacity. The attendant lung hyperinflation created difficulty in obtaining beat-by-beat measurements of LVSV via Doppler flowmetry of the ascending aorta. Therefore, we obtained LVSV measurements using finger photoplethysmography and the Modelflow algorithm [33]. It was not feasible to calibrate the Finometer against a ‘gold-standard’ measure of CO in our study and, consequently, the absolute magnitudes of the haemodynamic parameters reported herein may be inaccurate [53]. This limitation was circumvented by using a repeated-measures design, where the errors introduced from uncalibrated Modelflow simulations are accounted for on a subject-by-subject basis. In addition to the above concerns, the physical constraints imposed by the Doppler probe headset precluded the measurement of cerebral tissue oxygenation via near-infrared spectroscopy [9].

Finally, the present study examined the impact of involuntary respiratory contractions on the cerebral haemodynamic response to maximal apnoea in trained divers under “dry” laboratory conditions. Therefore, we caution that our findings may not readily generalise to untrained/naïve participants, or to the conditions imposed on subjects when breath holding under water at depth. On this latter point, it is currently not feasible to use transcranial Doppler ultrasound to measure CBFV under water. Future studies may overcome these limitations via computational modelling of the pressure-flow characteristics of peripheral and cerebral vessels [54]–[56].

## Summary of Findings

The findings of our study demonstrate that involuntary respiratory contractions play an important, yet complex role in mediating the CBFV response to maximal breath holding. On the one hand, involuntary respiratory contractions seemingly enhance central and cerebral haemodynamics during the struggle phase. While on the other hand, these contractions affect marked respiratory variability in cerebral blood flow and, presumably, O_2_ delivery to the brain. Most importantly, our data indicates that larger amounts of respiratory variability in cerebral blood flow during the struggle phase negatively impacts on breath holding performance.
